# Multiple origins, one evolutionary trajectory: gradual evolution characterizes distinct lineages of allotetraploid *Brachypodium*

**DOI:** 10.1093/genetics/iyac146

**Published:** 2022-10-11

**Authors:** Virginia T Scarlett, John T Lovell, Mingqin Shao, Jeremy Phillips, Shengqiang Shu, Joanna Lusinska, David M Goodstein, Jerry Jenkins, Jane Grimwood, Kerrie Barry, Boulos Chalhoub, Jeremy Schmutz, Robert Hasterok, Pilar Catalán, John P Vogel

**Affiliations:** U.S. Dept. of Energy Joint Genome Institute, Berkeley, CA 94720, USA; Department of Plant and Microbial Biology, University of California, Berkeley, Berkeley, CA 94720, USA; Genome Sequencing Center, HudsonAlpha Institute for Biotechnology, Huntsville, AL 35806, USA; U.S. Dept. of Energy Joint Genome Institute, Berkeley, CA 94720, USA; U.S. Dept. of Energy Joint Genome Institute, Berkeley, CA 94720, USA; U.S. Dept. of Energy Joint Genome Institute, Berkeley, CA 94720, USA; University of Silesia in Katowice, 40-007 Katowice, Poland; U.S. Dept. of Energy Joint Genome Institute, Berkeley, CA 94720, USA; Genome Sequencing Center, HudsonAlpha Institute for Biotechnology, Huntsville, AL 35806, USA; Genome Sequencing Center, HudsonAlpha Institute for Biotechnology, Huntsville, AL 35806, USA; U.S. Dept. of Energy Joint Genome Institute, Berkeley, CA 94720, USA; Agroscope, 1260 Nyon, Switzerland; U.S. Dept. of Energy Joint Genome Institute, Berkeley, CA 94720, USA; Genome Sequencing Center, HudsonAlpha Institute for Biotechnology, Huntsville, AL 35806, USA; University of Silesia in Katowice, 40-007 Katowice, Poland; University of Zaragoza, 22071 Huesca, Spain; U.S. Dept. of Energy Joint Genome Institute, Berkeley, CA 94720, USA; Department of Plant and Microbial Biology, University of California, Berkeley, Berkeley, CA 94720, USA

**Keywords:** *Brachypodium*, polyploidy, genomics, fractionation, genome dominance, structural variation, transposable element dynamics, Plant Genetics and Genomics

## Abstract

The “genomic shock” hypothesis posits that unusual challenges to genome integrity such as whole genome duplication may induce chaotic genome restructuring. Decades of research on polyploid genomes have revealed that this is often, but not always the case. While some polyploids show major chromosomal rearrangements and derepression of transposable elements in the immediate aftermath of whole genome duplication, others do not. Nonetheless, all polyploids show gradual diploidization over evolutionary time. To evaluate these hypotheses, we produced a chromosome-scale reference genome for the natural allotetraploid grass *Brachypodium hybridum*, accession “Bhyb26.” We compared 2 independently derived accessions of *B. hybridum* and their deeply diverged diploid progenitor species *Brachypodium stacei* and *Brachypodium distachyon*. The 2 *B. hybridum* lineages provide a natural timecourse in genome evolution because one formed 1.4 million years ago, and the other formed 140 thousand years ago. The genome of the older lineage reveals signs of gradual post-whole genome duplication genome evolution including minor gene loss and genome rearrangement that are missing from the younger lineage. In neither *B. hybridum* lineage do we find signs of homeologous recombination or pronounced transposable element activation, though we find evidence supporting steady post-whole genome duplication transposable element activity in the older lineage. Gene loss in the older lineage was slightly biased toward 1 subgenome, but genome dominance was not observed at the transcriptomic level. We propose that relaxed selection, rather than an abrupt genomic shock, drives evolutionary novelty in *B. hybridum*, and that the progenitor species’ similarity in transposable element load may account for the subtlety of the observed genome dominance.

## Introduction

Nearly all plant lineages have experienced at least 1 polyploidy event, or whole genome duplication (WGD), in their recent or ancient past (Clark and Donoghue 2018). Today’s diploids have undergone a process known as genetic diploidization, in which a polyploid loses genomic sequence over evolutionary time and becomes diploid again, though some duplicate genes are retained (Ma and Gustafson 2005). Polyploidy is an important source of genetic novelty and contributes to adaptive evolution (Van de Peer *et al.* 2017, 2021; Baduel *et al.* 2018).

In many cases, WGD is accompanied by rapid genome restructuring, in line with the hypothesis that WGD may represent a kind of “genomic shock” (McClintock 1984). The term “genomic shock” has been formally defined as a hybridization event “that induces a series of rapid genetic and epigenetic modifications as a result of conflicts between parental genomes” (Bird *et al.* 2018), though in practice “genomic shock” is often used in to indicate any sort of dramatic genetic consequence of hybridization or of WGD. The most dramatic examples of genomic shock are chromosomal rearrangements resulting from recombination between homeologous or homologous chromosomes, which may occur in the early generations after WGD (Ramsey and Schemske 2002) in allopolyploids (those whose progenitors are different species) and autopolyploids (those whose progenitors are from the same species), respectively (Grandont *et al.* 2013). Homeologous rearrangements are common in resynthesized polyploids (Mason and Wendel 2020), and evidence for them has been observed in a number of natural polyploids including *Brassica napus* (Chalhoub *et al.* 2014; Hurgobin *et al.* 2018), cotton (Guo *et al.* 2014) domesticated strawberry (Edger *et al.* 2019), quinoa (Jarvis *et al.* 2017), peanut (Bertioli *et al.* 2019), *Perilla frutescens* (Zhang *et al.* 2021), and the neoallopolyploid *Tragopogon miscellus* (Chester *et al.* 2012).

Some allopolyploids exhibit a dominant subgenome, whose genes are expressed at higher levels than their homeolog(s) on the other subgenome(s) (Alger and Edger 2020). It remains unclear to what extent genome dominance is established instantaneously or gradually. The evidence suggests both: expression bias established in the early generations following WGD may be reinforced over evolutionary time, with the dominant subgenome ultimately contributing more genes to the fully diploidized genome (Flagel *et al.* 2008; Feldman and Levy 2009; Flagel and Wendel 2010; Woodhouse *et al.* 2014; Edger *et al.* 2017).

Transposable element (TE) activation (transcription and/or transposition) can also occur following WGD on short and long timescales. Post-WGD epigenetic changes are not uncommon in polyploids (Ha *et al.* 2009; Parisod *et al.* 2009; Kenan-Eichler *et al.* 2011; Yaakov and Kashkush 2012; Yuan *et al.* 2020; Jiang *et al.* 2021). In allopolyploids, a single TE family or several families may be activated immediately following WGD, probably due to epigenetic incompatibilities between subgenomes (Madlung *et al.* 2005; Parisod *et al.* 2009; Martienssen 2010; Groszmann *et al.* 2011; Yaakov and Kashkush 2012; Sarilar *et al.* 2013; Gantuz *et al.* 2022). TE movement can also occur in polyploids over longer timescales due to relaxed selection because duplicate genes allow for a greater tolerance for TE insertions (Ågren *et al.* 2016; Baduel *et al.* 2019).

While some polyploids show chromosome rearrangements, expression dominance, and TE activation following WGD, these responses are not universal. Many natural allopolyploids show little to no genome restructuring, including *Arabidopsis suecica* (Burns *et al.* 2021), *Eragrostis teff* (VanBuren *et al.* 2020), *Capsella bursa-pastoris* (Douglas *et al.* 2015), and white clover (Griffiths *et al.* 2019). Thus, while WGD is often regarded as a profound genomic shock, a number of species seem to contradict this paradigm. The plant response to WGD is controlled by several complex factors including meiosis-related genes (Grandont *et al.* 2013), progenitor divergence (Ramsey and Schemske 2002), TE abundance or TE load (Woodhouse *et al.* 2014; Wendel *et al.* 2018), and demographic factors (Steige and Slotte 2016). Given the complexity of the plant response to WGD, simple model organisms are needed to reveal how the characteristics of the progenitor species’ genomes may predispose a polyploid to a particular evolutionary trajectory.

*Brachypodium hybridum* (2*n* = 4*x* = 30) is an annual allotetraploid grass that is native to the Mediterranean region but has spread all over the world, surpassing the range of either of its diploid progenitors, *Brachypodium stacei* (2*n* = 2*x* = 20), and the well-known model grass *Brachypodium distachyon* (2*n* = 2*x* = 10) (Catalán *et al.* 2012, 2016). We know that *B. hybridum* has multiple origins because some lines have chloroplasts that resemble the chloroplasts in *B. distachyon* (D-plastotype accessions), and other lines have chloroplasts that resemble the chloroplasts of *B. stacei* (S-plastotype accessions). Since chloroplasts are inherited from the maternal parent, the existence of distinct plastotypes indicates that *B. hybridum* must have arisen from more than 1 cross. Consistent with this hypothesis, the corresponding nuclear subgenomes of the 2 plastotypes show large evolutionary divergence from each other (Gordon *et al.* 2020). In a previous study (Gordon *et al.* 2020), we designated the accession Bhyb26 as the reference genome for the D-plastotype lineage and ABR113 as the reference genome for the S-plastotype lineage. Crosses between these 2 *B. hybridum* accessions resulted in sterile offspring, consistent with the lack of genetic evidence for hybridization between them (Gordon *et al.* 2020). The compact, naturally inbred genomes of these 2 polyploid lineages, their reproductive isolation, and the relative simplicity of the WGD make this system a valuable model for detailed study of polyploid genome evolution.

We previously demonstrated that *B. hybridum* ABR113, which corresponds to the type specimen of *B. hybridum* (Catalán *et al.* 2012), shows no sign of genome rearrangement nor of substantial gene loss (Gordon *et al.* 2020). A resynthesized *B. hybridum* line also bore no evidence of genomic rearrangements, based on a panel of SSR- and gene-derived PCR markers (Dinh Thi *et al.* 2016). This contrasts with some polyploid plants, such as *B. napus* (Szadkowski *et al.* 2010), tobacco (Lim *et al.* 2006), cucumber (Yu, Wang, *et al.* 2021), and certain wheats (Mirzaghaderi and Mason 2017) in which the first generation following WGD is genetically unstable, and meiosis may (Tian *et al.* 2010) or may not (Gou *et al.* 2018) stabilize over the first few generations. All *B. hybridum* lines examined so far show no sign of aneuploidy, homeologous exchange, nor chromosomal rearrangement (Dinh Thi *et al.* 2016; Gordon *et al.* 2020).

**Fig. 1.**
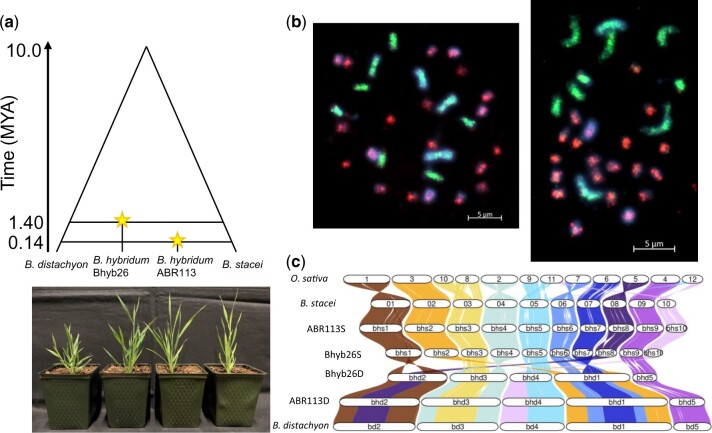
Both independent allopolyploid lineages reveal remarkable genome stability. a) Cladogram illustrating relationships in the *B. hybridum* polyploid complex. b) BAC-FISH with probes specific to either the S subgenome (8P20; red fluorescence) or D subgenome (ABR1-63-E6; green fluorescence) indicate the absence of large-scale rearrangement between subgenomes. Left, Bhyb26, right, ABR113. Blue fluorescence, DAPI. Bars, 5 µm. c) Riparian (synteny) plot showing high collinearity between each subgenome and its progenitor, and low collinearity between the polyploid subgenomes, consistent with the high divergence of the progenitor species’ genomes.

*B. hybridum* ABR113 formed roughly 140,000 years ago, making it a relatively “young” polyploid, so it was difficult to draw conclusions about its immediate diploidization. *B. hybridum* Bhyb26, on the other hand, formed 1.4 million years ago, meaning that this lineage has had substantially more time for evolution toward diploidization (Gordon *et al.* 2020) (Fig. 1a). Here, we present a high-quality PacBio-based reference genome for *B. hybridum* Bhyb26, and we perform an in-depth survey of its structure and TE landscape. The Bhyb26 genome, like the other *B. hybridum* genomes, reveals no convincing evidence of homeologous rearrangement. However, we did find evidence that Bhyb26, unlike the younger lineage, has experienced post-WGD structural change and slight but significantly biased gene loss. Remnants of these “lost” genes show signs of pseudogenization. We did not find evidence of increased TE proliferation, nor did we observe increased TE insertion in or near genes, a mechanism by which TEs have been proposed to drive diploidization (Wendel *et al.* 2018). Therefore, TEs do not seem to be contributing to the observed gene loss. Our study demonstrates that polyploids with multiple origins can be effectively used to study polyploid evolution, serving—with some caveats—as natural replicates of the diploidization experiment.

## Materials and methods

### Sample preparation

For details on the lines used in this study and preparation of high-molecular weight DNA for PacBio sequencing, see Gordon *et al.* (2020). PacBio sequencing was performed on a PacBio RSII instrument at the HudsonAlpha Institute.

### RNA-Seq

To collect leaf tissue, plants were grown in a growth chamber in short-day conditions (26°C 10 h light, 18°C 14 h dark). Leaf tissues were harvested from plants at the 4–5 leaf stage. To collect spikelets, plants were grown in long-day conditions (26°C 16 h light, 18°C 8 h dark). Spikelets were harvested 3 days after inflorescence emergence. For root tissue, plants were grown in plastic sundae cups with lids on sterile MS medium, and roots were harvested at 1–2 weeks. Callus tissue was prepared as described (Bragg *et al.* 2015). RNA was extracted using TRIzol (Invitrogen) and purified with the Purelink RNA Mini Kit (Invitrogen) including DNA removal with the Purelink DNAse Set (Invitrogen). Stranded short-read RNA-seq libraries were created using the Illumina TruSeq kit and quantified by qPCR. Sequencing was performed using an Illumina NovaSeq S4 instrument. Stranded long-read RNA-seq libraries were created using the PacBio SMRTbell Template Prep Kit v. 1.0 with or without 2–10-kb size selection using the BluePippin system (Sage Science). Sequencing was performed using a PacBio Sequel II instrument.

Raw RNA-seq reads were filtered and trimmed using BBDuk from the BBtools package (https://sourceforge.net/projects/bbmap). Reads were aligned to the complete reference genome (Bhyb26 v2.1) using BBmap. To increase mapping stringency, given the redundancy of a polyploid genome, reads were required to share 90% sequence identity with the target location, and ambiguous reads were discarded. Gene-level counts were obtained using HTSeq (Anders *et al.* 2015). Transcripts per million (TPM) values were calculated using a custom Python script (https://github.com/vtartaglio/Scarlett_et_al_2022/blob/master/fig4/countsToTPMbasicNEW.py; last accessed 6-20-2022).

### Assembly

Assembly of the Bhyb26 genome was performed with MECAT (Xiao *et al.* 2017) and polished using ARROW (https://github.com/PacificBiosciences/GCpp; last accessed 6-20-2022).

Misjoins in the assembly were identified using Hi-C data as part of the JUICER pipeline (Durand, Shamim, *et al.* 2016). No misjoins were identified in the polished assembly. Scaffolds were then oriented, ordered, and joined together using Hi-C scaffolding. Significant telomeric sequence was properly oriented in the assembly. Hi-C reads were then aligned to the joined release. A contact map was generated using JUICER-pre and visualized using JUICEBOX (Durand, Robinson, *et al.* 2016) as a quality control check on the order/orientation of contigs in the chromosomes. Care was taken to ensure that telomeres were properly oriented in the chromosomes, and the resulting sequence was screened for retained adapter/vector and/or contaminants.

Adjacent alternative haplotypes were identified on the joined contig set. Althap regions were collapsed using the longest common substring between the 2 haplotypes. A total of 22 adjacent alternative haplotypes were collapsed. Chromosomes of the v.2 release were numbered and oriented relative to the previous v.1 release (Gordon *et al.* 2020). Finally, homozygous SNPs and INDELs were corrected using 40× of Illumina reads.

### Annotation

Transcript assemblies were made from Illumina RNA-seq reads using PERTRAN, which conducts genome-guided transcriptome short-read assembly via GSNAP (Wu and Nacu 2010) and builds splice alignment graphs after alignment validation, realignment, and correction. PacBio Iso-Seq circular consensus sequences (CCSs) were corrected and collapsed by a genome guided correction pipeline, which aligns CCS reads to the genome with GMAP (Wu and Nacu 2010) and clusters alignments when all introns are the same or 95% overlap for single exon. Subsequently 625,901 total transcript assemblies were constructed using PASA (Haas *et al.* 2003) from the Iso-seq transcript assemblies. Loci were determined by transcript assembly alignments and/or EXONERATE (Slater and Birney 2005) alignments of proteins from diverse plant species and Swiss-Prot proteomes to the repeat-soft-masked *B. hybridum* Bhyb26 genome using RepeatMasker (Smit *et al.* 2013–2015). Gene models were predicted by homology-based predictors, FGENESH+ (Salamov and Solovyev 2000), FGENESH_EST, and EXONERATE, PASA assembly ORFs (in-house homology constrained ORF finder) and from AUGUSTUS (Stanke *et al.* 2006) trained by the high confidence PASA assembly ORFs and with intron hints from short-read alignments. The best scored predictions for each locus were selected using multiple positive factors including EST and protein support, and 1 negative factor: overlap with repeats. The selected gene predictions were improved by PASA. Improvement included adding UTRs, splicing correction, and adding alternative transcripts. PASA-improved transcripts were selected based on Cscore, protein coverage, EST coverage, and their CDS overlapping with repeats. Weak gene models, incomplete gene models, gene models whose protein was more than 30% in Pfam TE domains, low homology supported without fully transcriptome supported gene models, and gene models consisting of a short single exon without protein domain nor good expression gene models were manually filtered out.

### BAC-FISH

BAC-FISH was performed on *B. hybridum* Bhyb26 and ABR113 with *B. distachyon*- and *B. stacei*-derived Bacterial Artificial Chromosome clones (BACs): ABR1-63-E6 from *B. distachyon* ABR1 genomic DNA (gDNA) library (Hasterok, Maasek, *et al.* 2006) and 08P20 from *B. stacei* gDNA library made by B. Chalhoub. After isolation using the standard alkaline lysis method, BAC DNAs were labeled by nick translation using digoxigenin-11-dUTP (ABR1-63-E6) or tetramethylrhodamine-5-dUTP (08P20) as previously described (Jenkins and Hasterok 2007). Chromosome preparations were made using the method of Hasterok, Dulawa, *et al.* (2006) and Gordon *et al.* (2020). After germinating seeds in Petri dishes on moist filter paper, seedlings were incubated for 24 h in ice-cold water and fixed in 3:1 methanol-glacial acetic acid. After excision, roots were enzymatically digested for 2 h at 37°C in 1% (w/v) cellulase (Calbiochem), 1% (w/v) cellulase “Onozuka R-10” (Serva), and 8% (v/v) pectinase (Sigma). After removing the root cap and skin, the digested meristematic material was transferred to a slide and squashed in a drop of 45% acetic acid. Coverslips were removed after freezing. Fluorescence in situ hybridization (FISH) followed the method of Jenkins and Hasterok (2007) with previously described modifications (Lusinska *et al.* 2018; Gordon *et al.* 2020). The hybridization mixture comprised 40% deionized formamide, 15% (w/v) dextran sulfate, 2× SSC, 0.5% SDS and BAC DNA probes, each at the final concentration of 75–200 ng/slide. The probes in the hybridization mixture were predenatured at 80°C for 10 min and, after application to the preparations, denatured at 70°C for 4.5 min. Hybridization was carried out at 37°C in a humid chamber for at least 16 h. Posthybridization washes were carried at the equivalent of ∼60% stringency, and the digoxigenated probe was immunodetected using relevant (FITC-conjugated anti-digoxigenin, Roche) antibodies. The preparations were counterstained with 2.5 µg/ml DAPI, mounted in Vectashield (Vector Laboratories) and analyzed under Axioimager.Z.2 epifluorescent microscope (Zeiss) coupled with AxioCam Mrm high-sensitivity monochromatic camera (Zeiss).

### Synteny and gene loss

GENESPACE v.0.9.4 (Lovell *et al.* 2018, 2022) (https://github.com/jtlovell/GENESPACE) was run with default parameters to evaluate synteny among *Brachypodium* genomes and rice (*B. distachyon* Bd21 v3.2, proteome id: 556; *B. stacei* v1.1, proteome id: 316; *B. hybridum* ABR113 v1.1, proteome id: 463; *B. hybridum* Bhyb26 v.2.1, proteome id: 693; *Oryza sativa* MSU v0.7, Phytozome, proteome id: 323). All reference genomes were obtained from Phytozome (Goodstein *et al.* 2012) (https://phytozome-next.jgi.doe.gov/; last accessed 6-20-2022). GENESPACE infers orthology relationships among primary peptide sequences using orthofinder (Emms and Kelly 2019) but limits the search to colinear (syntenic) blocks identified by MCScanX (Wang *et al.* 2012). GENESPACE output includes syntenic dotplots and riparian plots, which were used to visually assess structural variation, and groups of orthologous genes (orthogroups), which were the basis of the gene loss analysis. We examined dot plots based on ortholog similarity alone as well as similarity plus physical position. Individual chromosomes that appeared to contain rearrangements were further validated using Gepard (Krumsiek *et al.* 2007), which builds dotplots from k-mers rather than genes. In all cases, the Gepard and GENESPACE results were essentially identical.

Our procedure for pseudogene identification was essentially that of Gordon *et al.* (2020), except that we started with incomplete orthogroups rather than incomplete gene triplets. The neighborhood of the “missing gene” in Bhyb26 was identified based on orthology relationships of 10 genes flanking, or nearly flanking, the diploid gene from the progenitor corresponding to the subgenome with the missing gene. The protocol was as follows: once we had identified the diploid gene corresponding to the missing Bhyb26 gene, we “walked” outward along the diploid chromosome in both directions, checking whether each nearby gene had a single ortholog in the appropriate Bhyb26 subgenome. If a gene had no orthologs or many orthologs, it was skipped and we proceeded to the next-closest gene. This process was repeated until we had 10 informative genes flanking the original diploid gene, 5 on each side. The syntenic orthogroup was discarded if we had to check more than 25 genes on the one side, or if we ran off the chromosome before we had 5 good neighbors. At this point, 588 of our original 664 orthogroups remained. Next, we required that at least 4 of the 5 neighboring genes on either side of the original diploid genes had orthologs in the same 200 kb region of the Bhyb26 genome. At this point, 534 orthogroups remained. Finally, we recorded the Bhyb26 orthologs of the upstream and downstream neighbors that were closest to the original diploid gene and extracted the region between and including these 2 “anchor” genes. If the region was greater than 20 kb, the orthogroup was discarded. Finally, 517 candidate Bhyb26 regions remained. See https://github.com/vtartaglio/Scarlett_et_al_2022/tree/master/fig3; last accessed 6-20-2022.

Once we had identified the Bhyb26 genomic region potentially containing the missing gene, the region was extracted using bedtools (Quinlan 2014). The diploid peptide was then aligned to that region using the codon- and intron-aware protein2genome model of EXONERATE (Slater and Birney 2005). We found that these EXONERATE alignments were of excellent quality, but EXONERATE codon-aware DNA–DNA alignments were of poor quality, especially on long genes containing frameshifts. Therefore, we next aligned the diploid coding sequence (from Phytozome) to the inferred Bhyb26 coding sequence (from EXONERATE) using MACSE (Ranwez *et al.* 2011, 2018), and these alignments were used to calculate pairwise nonsynonymous to synonymous substitution rate ratios via the yn00 program from PAML (Yang 2007). The same procedure was applied to fully conserved Bhyb26 genes as a control, with each of 1,000 trials consisting of 224 BhD genes and 240 BhS genes (464 total), since this was the final number of aligned “missing” genes from each subgenome.

### TE annotation and analysis

TE annotation was performed with an in-house pipeline. The pipeline was not designed for external use, but the scripts are available at https://github.com/vtartaglio/Scarlett_et_al_2022/tree/master/fig5; last accessed 6-202022. First, monocot TEs were pulled from the RepeatMasker database, and these were concatenated to the TREP database to create an initial TE library. To discover TEs from the *Brachypodium* genomes that are not in public databases, we ran a suite of TE discovery tools. Tools used were LTR-Harvest (Ellinghaus *et al.* 2008), LTR_retriever (Ou and Jiang 2018), TransposonPSI (http://transposonpsi.sourceforge.net/; last accessed 6-20-2022), MITE-Tracker (Crescente *et al.* 2018), and RepeatModeler2 (Flynn *et al.* 2019). These TEs were added to the library of publicly available repeats and redundancy was removed with CD-HIT (Fu *et al.* 2012) according to the “80-80-80 rule” (Wicker *et al.* 2007) (cd-hit-est -c 0.8 -G 0 -aS 0.8 -n 5 -T 0 -d 0 -M 0). Sequences were clustered if they had 80% identity locally, and the alignment had to cover at least 80% of the shorter sequence. Only the longest sequence (the representative sequence) from each cluster was retained. Representative sequences less than 80 bp were discarded. Next, ProtExcluder from the MAKER-P pipeline (Campbell *et al.* 2014) was used to search the TE library against a plant protein database, and TEs with significant hits to genes were removed. The result of this process was a nonredundant library containing TE exemplars from a variety of monocots and a de novo TE exemplars from that *Brachypodium* genome. Each genome (*B. distachyon* Bd21, *B. distachyon* Bd1-1, *B. stacei* ABR114, *B. hybridum* ABR113, and *B. hybridum* Bhyb26) had its own separate TE library.

All the genomes listed above were annotated with RepeatMasker (Smit *et al.* 2013–2015) using the appropriate TE library. Noncontiguous genomic sequences that match the same exemplar were designated fragments of a single TE copy if certain distance and orientation criteria were met, using “one code to find them all” (Bailly-Bechet *et al.* 2014) with default parameters. A TE family was defined as the set of all TE copies that were hits to a particular exemplar. Subgenome-specific TE families were defined as those that had at least 5 members and that had at least 90% of their copies on one of the 2 subgenomes (this latter criterion comes from Wicker *et al.* (2018)).

TEMP2 (Yu, Huang, *et al.* 2021) was used to identify TE polymorphisms relative to the ABR113 reference genome. Library quality was assessed with FASTQC (https://www.bioinformatics.babraham.ac.uk/projects/fastqc/; last accessed 6-20-2022). Short-read libraries were the same as those used in (Gordon *et al.* 2020). Only transposon insertion polymorphisms (TIPs) that were supported by reads on both ends (“1p1”) and that had a frequency of 20%—that is, at least 20% of sequenced genome supports the insertion—were considered.

To estimate the insertion times of intact LTR-RTs, we largely followed the method of (Wicker *et al.* 2018), which itself derives from (SanMiguel *et al.* 1998). The 3′ and 5′ LTRs of individual LTR-RTs were aligned to each other with MAFFT (Katoh *et al.* 2002) (einsi –adjustdirectionaccurately) and trimmed with trimAl (Capella-Gutiérrez *et al.* 2009) (trimal -gapthreshold 0.8). Then, EMBOSS distmat (http://emboss.sourceforge.net/; last accessed 6-20-2022) was run on each alignment with the Kimura 2-parameter correction (distmat -nucmethod 2) to obtain the % identity between the LTRs. Insertion time was calculated with the equation: *T* = *D*/2*t*, where *T* is the time elapsed since the insertion, *D* is the estimated LTR divergence, and *t* is the substitution rate, for which we used 1.3 × 10^−8^ substitutions per site per year (Ma and Bennetzen 2004).

## Results

### Assembly and annotation

We assembled a chromosome-scale reference genome of the naturally inbred allotetraploid *B. hybridum* accession Bhyb26, which was collected in the wild in Jaen, Spain. In a previous study, we built an Illumina-based genome assembly (Bhyb26 v1.1) (Gordon *et al.* 2020). The new genome assembly (Bhyb26 v2.1) was constructed de novo using PacBio and Hi-C technologies. The main assembly was performed with MECAT (Xiao *et al.* 2017) using 45× consensus long-read coverage (average read length of 19,692 bp), and the resulting assembly was polished with 40× Illumina reads using Arrow (https://github.com/PacificBiosciences/GCpp; last accessed 6-20-2022). Hi-C scaffolding was performed using the Juicer pipeline (Durand, Shamim, *et al.* 2016). There were 61 contigs with a contig N50 of 16.5 Mb. A total of 51 joins were applied to the broken assembly to form the final assembly consisting of 32 scaffolds. Fifteen of the 32 scaffolds contain 99.69% of the assembled sequence, and these correspond to the 15 chromosomes of *B. hybridum* (5 BhD and 10 BhS). The remaining 17 scaffolds totaled about 1.6 Mb of sequence. The final genome size is 528.5 Mb and contains less than 0.1% gaps.

Annotation was performed with the JGI pipeline (see *Materials and Methods*). Transcript assemblies were made from ∼290 million 2 × 150 stranded paired-end Illumina RNA-seq reads and 23 million PacBio Iso-Seq CCSs, each generated from 4 tissues: leaf, spikelet, root, and callus. The annotation (v.2.1) contains 53,864 primary transcripts with an average of 5.1 exons, a median exon length of 166 bp, and a median intron length of 142 bp. The BUSCO v3.0.2 score on Embryophyta odb9 is 99.7% complete.

### Synteny and structural variation

We began our investigation with a survey of Bhyb26 genome structure using molecular cytogenetics. FISH experiments with BACs containing large gDNA inserts as probes (BAC “landing”) (Jenkins and Hasterok 2007) were conducted using 2 clones from previously constructed BAC libraries (Hasterok, Marasek, *et al.* 2006; Chalhoub B, personal communication) (Fig. 1b). The BAC ABR1-63-E6 containing *B. distachyon* gDNA was found to reliably hybridize with the entire D subgenome, but it did not hybridize with any chromosomes of the S subgenome. The BAC 8P20 containing *B. stacei* gDNA hybridized with the entire S subgenome chromosomes, but not with the D subgenome chromosomes. These 2 BACs discriminated between subgenomes in both polyploids. Therefore, in both Bhyb26 and ABR113, the subgenomes are readily distinguishable at the level of molecular probes, and no evidence of sequence exchange between subgenomes was observed.

Next, we performed a computational survey of Bhyb26 genome structure. Syntenic blocks between each subgenome and its diploid progenitor species were identified using the GENESPACE pipeline (Lovell *et al.* 2018, 2022) (Fig. 1c). 97.9% of the Bhyb26 D subgenome was contained within blocks syntenic to *B. distachyon* (Bd21 v3.2), and 93.4% of the Bhyb26 S subgenome was contained within blocks syntenic to *B. stacei* (ABR114 v1.1). There were 41 Bd-BhD syntenic blocks and on average they were 6.6 Mb in length, while the 124 Bs-BhS syntenic blocks were on average 2 Mb in length. This lower concordance between the S subgenome and its progenitor species may be attributable to the *B. stacei* reference genome being a lower-quality Illumina assembly, and may not reflect biological divergence.

**Fig. 2.**
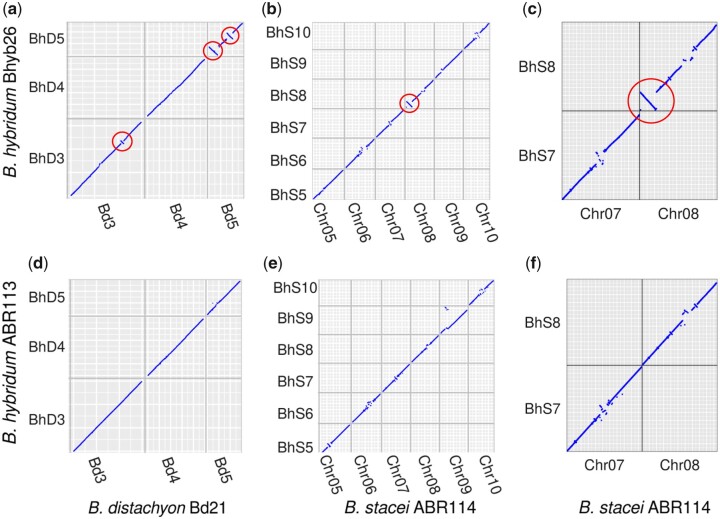
Inversions in Bhyb26. Synteny-constrained dot plots relative to diploid progenitors revealed ∼2–7 Mb inversions (circled) on both subgenomes of Bhyb26, but similar structural variation was absent from ABR113. For visual clarity, not all chromosomes are shown. a) Bhyb26 D vs. *B. distachyon* Bd21, chromosomes 3–5. b) Bhyb26 S vs. *B. stacei* ABR114, chromosomes 5–10. c) Bhyb26 S vs. *B. stacei* ABR114, chromosomes 7 and 8. d–f), same as (a)–(c), but ABR113 instead of Bhyb26.

The synteny results revealed several inversions in Bhyb26 relative to its diploid progenitors (Fig. 2, a–c). On the D subgenome, there is a ∼2.3-Mb inversion on chromosome BhD3, as well as one ∼5-Mb and another ∼7-Mb inversion on chromosome BhD5 (Fig. 2a). On the S subgenome, there is a ∼4.2-Mb inversion at the top of chromosome BhS8, as well as smaller inversions (<1 Mb) on chromosomes BhS5 and BhS9 (Fig. 2b). We also ran our synteny pipeline on each *B. hybridum* ABR113 subgenome against the diploid progenitor species (Fig. 2, d–f). The *B. stacei* reference genome is much lower quality than the *B. distachyon* reference genome, and small inversions were common in the centromeres of the *B. stacei* dot plots (Fig. 2, b and e). The most prominent of these were on BhS3, BhS5, and BhS7. The inversions on BhS3 and BhS7 were not well-supported upon closer inspection of the dot plots (Fig. 2, c and f). The inversion that is apparently private to ABR113 on BhS5 was more clear, that is, all anchor genes in that region supported the inversion. However, given that it is in a centromere that contains small inversions in all 3 dot plots, it is likely that this is an assembly error. Thus, none of the inversions on the ABR113 S subgenome appear to reflect true structural variation.

To ascertain whether inversions or deletions are common between diploid *Brachypodium* accessions, we re-ran our synteny pipeline on 2 more long-read *B. distachyon* genomes, 1 from each of the 3 major populations of *B. distachyon*: Bd21 representing the Turkish+ clade, Bd30-1 representing the Spanish+ clade, and Bd1-1 representing the extremely delayed flowering+ (EDF+) clade (Gordon *et al.* 2017). We detected no inversions among these genomes. These results indicate that the Bhyb26 genome contains several inversions that are private to that lineage, and such inversions are not common among diverged *B. distachyon* accessions. While it is still possible that these inversions were present in the actual progenitors of Bhyb26 prior to polyploidization, the absence of any similarly dramatic structural variation in the widely sampled natural diversity of *B. distachyon* suggests that these inversions may well have occurred postpolyploidy.

### Gene loss

**Fig. 3.**
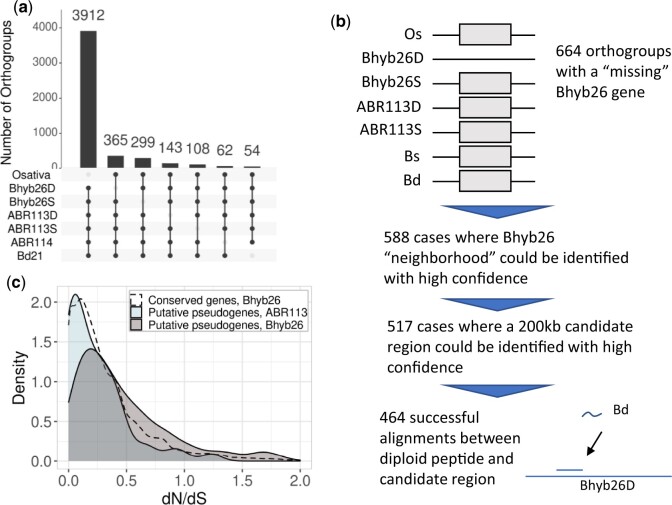
Bhyb26 shows more gene loss than ABR113. a) UpSet plot of orthogroups (groups of orthologous genes) reveals a high number of cases where a single Bhyb26 subgenome lacks an ortholog of an otherwise widely conserved gene. b) Workflow for identifying putative pseudogenes. c) Distribution of dN/dS ratios for Bhyb26 and ABR113 “lost genes,” and Bhyb26 widely conserved genes. All dN/dS values are relative to the corresponding diploid ortholog.

In a previous analysis (Gordon *et al.* 2020), we ascertained that the subgenomes of Bhyb26 were more genetically diverged from the corresponding diploid progenitor species’ reference genomes than were the subgenomes of ABR113, but the low-quality Bhyb26 assembly did not permit in-depth analysis of this variation. We were particularly interested in the degradation or loss of genes, which would be indicative of the early stages of diploidization. However, identifying genes that have been lost in Bhyb26 since WGD is difficult without its *true* progenitors, since gene presence–absence variation would be common among arbitrary accessions of *B. distachyon* and *B. stacei* (Gordon *et al.* 2017). We therefore searched for losses of highly conserved genes, reasoning that any gene that is conserved within and beyond the genus *Brachypodium* was probably present in the true progenitors of Bhyb26. Using the synteny and homology-based pipeline GENESPACE (Lovell *et al.* 2018), we identified 15,217 orthogroups that contained at least 1 orthologous gene in both subgenomes of both polyploids, each diploid genome, and rice (*O. sativa*). In other words, we identified many thousands of genes that are widely conserved across the genus *Brachypodium* and in rice. We then identified orthogroups where all but 1 genome or subgenome was represented (Fig. 3a). Unsurprisingly, orthogroups that had an ortholog in every *Brachypodium* sub/genome but not rice were most common (3,912 orthogroups). More surprisingly, the number of cases where a gene was “missing” from each *B. hybridum* genome was greater than we would expect by summing the progenitors, and this discrepancy was greater for Bhyb26 than for ABR113. 365 and 299 genes were present in every sub/genome except Bhyb26D and Bhyb26S, respectively; meanwhile, only 143 and 108 genes were present in every sub/genome except ABR113D and ABR113S, respectively. In other words, we identified only 251 “missing” genes in ABR113, but we found 664 in Bhyb26, a significant difference considering the number of genes in each genome (Pearson's Chi-squared test with Yates' continuity correction *P* = 4.84e−70). The high number of conspicuously absent genes in Bhyb26 suggests that at least some of these genes may be true pseudogenes or deletions that occurred post-WGD as a consequence of relaxed selection.

We interrogated these “missing genes,” which we dubbed putative pseudogenes, more closely (Fig. 3b). Six hundred sixty-four broadly conserved genes were absent in 1 Bhyb26 subgenome. In 517 of those cases, we were able to definitively identify a region of the Bhyb26 genome where the missing gene “should” be (see *Materials and Methods*). A total of 464 of those 517 regions contained sequence that could be aligned to the peptide sequence of the corresponding diploid gene (Supplementary Fig. 1). These sequences were scattered throughout the Bhyb26 genome (Supplementary Fig. 2a). As a control, we ran the same procedure on a set of 464 randomly selected Bhyb26 genes that were completely conserved, that is, genes from orthogroups in which all genomes are represented (see *Materials and Methods*). We repeated the random sampling and the analysis for a total of 1,000 trials. The putative pseudogenes were shorter than expected: for conserved genes, the alignments were 22.7 bp shorter than the peptide on average, while for the putative pseudogenes, they were 224.3 bp shorter (Table 1). In none of the 1,000 samples of conserved genes did this value exceed that of the putative pseudogenes. In addition, 18.8% of the Bhyb26 putative pseudogenes contained a premature termination codon (PTC), while none of the alignments between a fully conserved Bhyb26 gene and its diploid ortholog contained a PTC. The putative pseudogenes were also enriched for nonexpressed genes, defined as those with a TPM value of zero (Supplementary Fig. 2b). The expected frequency of nonexpressed genes was calculated for each of 4 tissues: root, leaf, floret, and callus, based on the sampled conserved genes. In all tissues, the frequency of nonexpressed genes among the putative pseudogenes was higher than expected for that tissue (one-sided exact binomial test: *P* = 5.93e−78, *P* = 1.43e−69, *P* = 1.72e−79, and *P* = 1.40e−41, respectively). Finally, a valid d*N*/d*S* ratio, that is, the ratio of nonsynonymous to synonymous amino acid substitutions (Yang 2007), could be calculated for 423 of the 464 genes (Fig. 3c). The average d*N*/d*S* for the lost genes was 0.53 as opposed to the conserved genes’ 0.36. This is a significant difference (Welch 2 sample *t*-test *P* = 2.1e−9) and is consistent with the hypothesis that the putative pseudogenes are experiencing relaxed selection. Out of 1,000 trials, there were no cases in which the average d*N*/d*S* from conserved genes exceeded the average d*N*/d*S* from the putative pseudogenes. Interestingly, when we repeated this procedure on the ABR113 “missing” genes, obtaining 220 putative pseudogenes and 106 d*N*/d*S* values (many of the alignments had no substitutions in the polyploid), the mean d*N*/d*S* for these putative pseudogenes was only 0.324, and their distribution was also similar to that of the conserved genes (Fig. 3c). Thus, only the Bhyb26 genes showed signs of pseudogenization. The putative pseudogenes went unannotated due to lack of homology, incidence of premature stop codons, and weak transcriptome support (Table 1).

**Table 1.** Characteristics of Bhyb26 putative pseudogenes vs. Bhyb26 annotated, conserved genes.

Metric	Putative pseudogenes	Conserved genes (average of 1,000 trials)
Mean length difference between Bhyb26 alignment and diploid ortholog in progenitor species genome (bp)	224.3	22.7
Percentage of alignments that contained a premature stop codon in Bhyb26 relative to diploid progenitor genome gene	18.8	0.0
Mean d*N*/d*S* (aligned to diploid progenitor genome ortholog)	0.53	0.36
Median transcripts per million (TPM)	0.0	4.14

We hypothesized that TE insertion into the gene body may have contributed to the inactivation of these putative pseudogenes. A total of 130 of the 464 putative pseudogenes (28%) contained a TE somewhere between the start and end of the alignable region. Meanwhile, in the 1,000 control trials, on average 196 of the 464 randomly selected conserved genes (42.2%) contained a TE (Supplementary Fig. 2c). There were no trials in which the number of putative pseudogenes containing a TE (130) exceeded the number of conserved genes containing a TE; therefore, the *P*-value of this one-sided test is zero. This shows that the putative pseudogenes are not more likely to contain a TE than we would expect by random chance, although it is still possible that TE insertions in nearby regulatory regions may have deactivated some of the genes.

Finally, we noticed that both polyploid lineages had apparently lost more genes from the D subgenome than the S subgenome (Fig. 3a). We performed a chi-square test to test whether the biased loss was significantly different from a bias we might expect by chance, based on the total number of genes in each subgenome. In Bhyb26, the difference was significant (Pearson's Chi-squared test with Yates' continuity correction *P* = 0.031), but not in ABR113 (*P* = 0.34). Together, all these results indicate that (1) a significant portion of the “missing” genes in Bhyb26 are of dubious functionality, (2) the gene loss is marked by small-scale substitutions and deletions rather than by rampant TE insertions or by deletion of entire genes, and (3) in Bhyb26, the S subgenome is slightly but significantly dominant in terms of gene retention.

### Gene expression

**Fig. 4.**
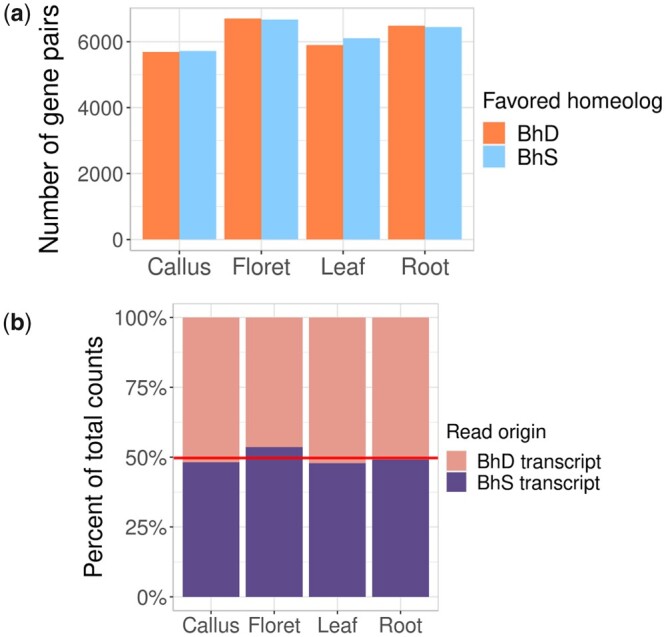
No expression bias in Bhyb26. a) Grouped bar chart showing the more highly expressed homeolog in gene pairs from 4 tissues. Only gene pairs where at least one homeolog had a TPM of >1.0 were considered. b) Stacked bar chart showing % of RNA-seq reads mapped to each subgenome. Horizontal line indicates the percentage of primary transcriptome base pairs that are from BhS transcripts.

Using Illumina RNA-seq data, we investigated whether 1 subgenome was systematically more highly expressed than the other in Bhyb26. Two analytical approaches were used: 1 for homeolog expression bias (HEB) and 1 for subgenome expression dominance. Since we did not have biological replicates, we could not conduct a formal HEB analysis, which requires accurate estimation of differential gene expression. Nevertheless, our experiment should be enough to distinguish a genome-wide trend, since the >50,000 genes provide a sort of replication, as do the 4 tissues sampled. We used GENESPACE (Lovell *et al.* 2018, 2022) to identify 1:1 homeologs between the subgenomes and then filtered out noisy gene pairs (those where both homeologs had a TPM < 1.0), and recorded whether the BhD homeolog or the BhS homeolog had the higher TPM. The chance that the homeolog from a particular subgenome had a higher TPM was near 50/50 in all tissues (Fig. 4a). The most extreme deviation from 50/50 was observed in leaf, in which 49.2% of gene pairs favor the BhD homeolog while 50.8% favor the BhS homeolog. To test whether the deviation from 50/50 was significant in any tissue, we performed an exact binomial test. Leaf was closest to significance (*P* = 0.052, alpha = 0.0125 with Bonferroni correction), but in no case was the pattern of HEB significantly different from what would be expected by random chance.

Finally, we checked for subgenome expression dominance, that is, evidence that the majority of expressed transcripts are coming from one of the 2 subgenomes. For this analysis, we worked with raw read counts rather than TPMs. To control for the fact that 1 subgenome may contribute more reads simply because it has more genes, we summed the lengths of the primary transcripts from all genes in each subgenome and took the total basepairs in each subgenome’s transcriptome to be our null expectation: 50.7% of counts would be expected to originate from BhD and 49.3% from BhS. All 4 tissues were close to this ratio, with floret being the most extreme deviation: 46.41% of counts were from BhD transcripts (Fig. 4b). While there may be some subtle BhS subgenome expression dominance in floret, there is no evidence for overall subgenome expression dominance in Bhyb26. This finding contrasts the above result for subtle BhS genome dominance in terms of gene retention.

### Gradual TE activity post-WGD

**Fig. 5.**
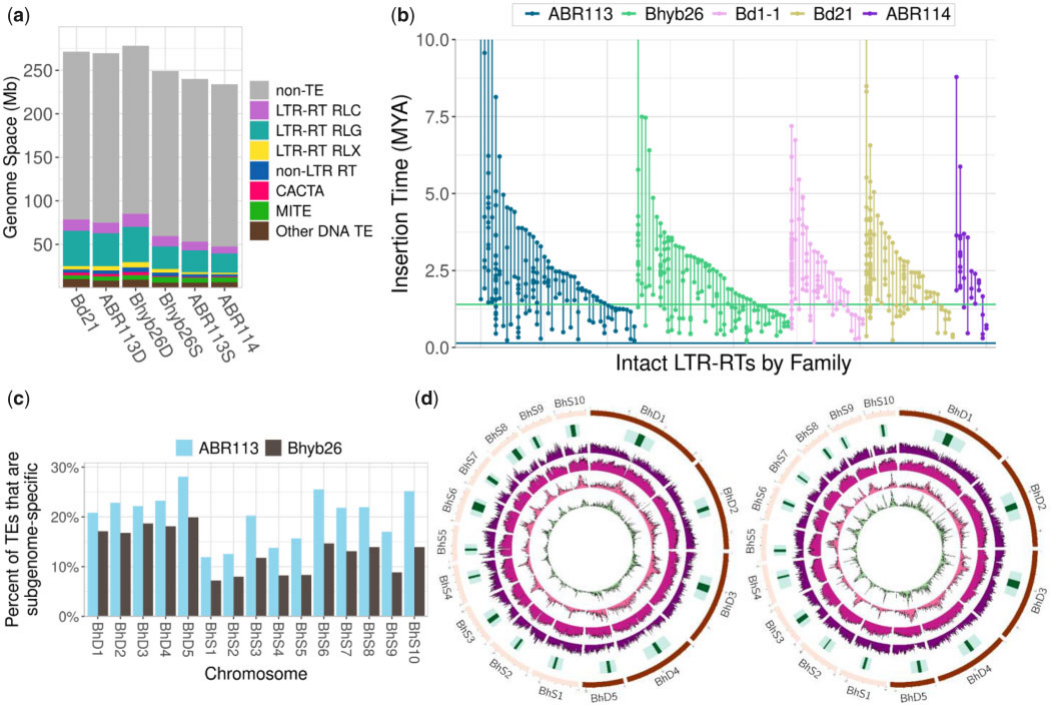
Gradual TE activity in *B. hybridum*. a) TE composition of sub/genomes by TE class. b) Insertion time analysis of intact LTR retrotransposons. Each vertical line is a TE family and each point is an individual TE copy. The length of the line indicates the lifespan of that LTR-RT family, or the difference in age between its oldest and youngest members. Horizontal lines denote WGD events for Bhyb26 (green; 1.4 MYA) and ABR113 (blue; 0.14 MYA). c) Subgenome-specific TEs, as a percentage of total TEs, per chromosome in the 2 polyploids. d) Overview of Bhyb26 (left) and ABR113 (right) genomes. Tracks, outer to inner: pericentromeres and centromeres, gene density, TE diversity, TE density, density of subgenome-specific TEs.

We annotated the TEs in *Brachypodium* genomes to see if disparities in TE content are driving biased genome evolution as has been observed in other polyploids (Woodhouse *et al.* 2014; Edger *et al.* 2017). Publicly available repeat sequences and de novo TEs were identified in 5 *Brachypodium* genomes (see *Materials and Methods*). The TE content of each polyploid subgenome was examined alone and compared to its progenitor species (Fig. 5a and Supplementary Table 1). The D sub/genomes (BhD of *B. hybridum* Bhyb26 and ABR113 and *B. distachyon* Bd21) were slightly more TE rich than the S sub/genomes (BhS of *B. hybridum* Bhyb26 and ABR113 and *B. stacei* ABR114). The Bhyb26 D subgenome was most TE rich at ∼31% TEs, while the *B. stacei* ABR114 genome was the most TE poor at ∼20% TEs. The latter figure may be an underestimate because the *B. stacei* genome is a short-read assembly, however, it is close to the Bhyb26 S TE content of ∼24%. The Bhyb26 S subgenome was enriched for full-length LTR-retrotransposons (LTR-RTs) relative to the other 2 S sub/genomes (122 vs. 108 and 64), which might be due to the long-read assembly. *Gypsy* (RLG) and *Copia* (RLC) elements (Wicker *et al.* 2007) occupied most of the TE space in all genomes. The ratio of *Gypsy* to *Copia* LTR-RT copies ranged from 1.16:1 to 1.30:1 in all genomes, but *Gypsy* elements were, on average, 1.36–3.01 times longer than *Copia* elements, so *Gypsy* elements constituted a much larger portion of the genome space than *Copia* elements (Fig. 5a and Supplementary File 1). Non-LTR retrotransposons also composed a substantial portion of the TE space, from 2.6 Mb in *B. stacei* to 5.4 Mb in Bhyb26-D (Supplementary File 1).

We surveyed TE polymorphisms among *B. hybridum* lines. We used the TE polymorphism detection software TEMP2 (Yu, Huang, *et al.* 2021) as implemented in the McClintock pipeline (Nelson *et al.* 2017) to quantify TE polymorphisms in short-read data from 20 *B. hybridum* lines, using ABR113 as our reference genome (Supplementary Fig. 3a). We focused on TIPs, that is, locations where a TE insertion was present in a resequenced genome but not in the reference. Bhyb26 had by far the greatest number of TIPs relative to ABR113. This increase was not due to sequencing technology because for all samples in this experiment, including Bhyb26, only raw Illumina reads were used. TIP number was not correlated with sequencing depth and only loosely correlated with library quality (*R*-squared = 0.34 for total TIPs vs. median per sequence quality score from FASTQC (https://www.bioinformatics.babraham.ac.uk/projects/fastqc/; last accessed 6-20, 2022)), so observed differences are unlikely to be due to sequencing artifacts. Interestingly, Bhyb118-5 is of the same plastotype as Bhyb26 but does not have nearly as many TIPs (Supplementary Fig. 3a). Previous phylogenetic analysis (Gordon *et al.* 2020) strongly suggests that Bhyb118-5 and Bhyb26 are of the same origin and neither is admixed with S-plastotype lineages of *B. hybridum*, so the greater number of polymorphisms in Bhyb26 suggests a possible uptick in TE activity since its divergence from Bhyb118-5. Many TE families contribute to TE diversity in *B. hybridum*. The 10 TE families that contribute the greatest number of TIPs are responsible for 52% of all TIPs (Supplementary Fig. 2a). The majority of TIPs came from *Gypsy* and *Copia* LTR-RTs, the most active classes of TE among *B. distachyon* accessions as well (Stritt *et al.* 2018, 2020) (Supplementary Fig. 3b). No single TE family contributed more than 25% of a genome’s total TIPs in any *B. hybridum* accession (Supplementary Fig. 3a). This is similar to what was observed in *B. distachyon*, where no single family dominates the TE diversity (Stritt *et al.* 2018).

Because LTR-RTs are among the most abundant and most active TEs in *B. hybridum* (Supplementary Table 1 and Supplementary Fig. 3b), we estimated insertion times for intact LTR-RTs in several *Brachypodium* genomes. Figure 5b shows, for each genome, all LTR-RT families that contain 2 or more intact copies. The number of intact LTR-RTs across our dataset appears to be a function of genome size and assembly quality. Neither polyploid shows an abundance of TE insertions at or after its WGD (Fig. 5b). All full-length ABR113 LTR-RTs pre-date the WGD. The percentage of full-length LTR-RTs less than 1.4 million years old was similar in ABR113 and Bhyb26: 40% and 44%, respectively. That Bhyb26 has slightly more “young” TEs again hints at the possibility of an uptick in TE activity since the WGD, though the difference is very slight.

We also looked for evidence of overall increased TE activity in and around genes in Bhyb26. We recorded the number of TEs that overlap a gene in each genome, requiring that the TE and gene be on the same strand, and UTR and intronic TEs were included. We found that 43% and 42% of Bhyb26 and ABR113 genes, respectively, overlap or contain a TE (Supplementary Table 1). In Bhyb26, 2.1% of exons overlap a TE, while in ABR113 5.1% of exons overlap a TE. TE overlap with genes also remained remarkably similar between the 2 polyploids when only TEs in either centromeres, pericentromeres, or distal regions were considered. The mean distance from a TE to a gene was similar in both polyploids: 1,272 bp in Bhyb26 and 1,289 bp in ABR113 (Supplementary Table 1). Thus, TEs in Bhyb26 show no elevated propensity to insert in or near genes compared to ABR113.

ABR113 had a slightly higher proportion of subgenome-specific TEs than Bhyb26 (Fig. 5, c and d, inner track). Subgenome-specific TEs were defined as TE copies belonging to a TE family consisting of at least 5 members and in which >90% of members were located in one of the 2 subgenomes (see *Materials and Methods*). 11.4% and 16.8% of all TE copies in Bhyb26 and ABR113, respectively, were from subgenome-specific TE families. This difference is slight, but the trend was consistent across chromosomes (Fig. 5c), and a paired *t*-test of the chromosome-level percentages was highly significant (*P* = 2.722 × 10^−8^). This slight but significant difference suggests that there has been some small-scale transfer of TEs between subgenomes in Bhyb26 post-WGD. Considering that Bhyb26 has a high number of TIPs relative to ABR113, it has a slightly higher proportion of “young” (<1.4 MY) LTR-RTs than ABR113 has, and the Bhyb26 TE fraction is slightly depleted for subgenome-specific TEs relative to that of ABR113, moderate post-WGD TE activity and exchange between subgenomes seems plausible. However, the possibility that the TE landscapes of the true progenitors of Bhyb26 were more similar to each other than those of ABR113 cannot be ruled out.

## Discussion

Our results indicate that diploidization is progressing slowly in *B. hybridum*, an allotetraploid with multiple origins. In contrast to the more recent *B. hybridum* lineage ABR113, the older Bhyb26 lineage shows several megabase-scale inversions and a greater extent of pseudogenization. In both lineages, gene loss slightly favored retention of genes in the S subgenome, though the difference was significant only in the older line, while in neither line was genome dominance supported by expression data. Finally, we found evidence for gradual rather than instantaneous post-WGD TE activity. We argue that these genomic changes were most likely made possible by relaxed selection post-WGD. The changes are modest overall, consistent with gradual post-WGD evolution.

The chromosomal rearrangements observed in Bhyb26 are not characteristic of homeologous exchange, a classic genomic shock response. Homeologous recombination can lead to duplications, deletions, and translocations (Mason and Wendel 2020). Inversions, on the other hand, more likely result from ectopic recombination or nonhomology directed DNA repair within a single chromosome. The inversions that are unique to Bhyb26 probably did not arise through homeologous exchange, so they could have occurred either pre- or post-WGD. However, we find no megabase-scale inversions between diverse accessions of the well-sampled diploid progenitor species *B. distachyon*. Thus, the available evidence suggests that such large inversions are not typical of intraspecific variation within *Brachypodium* diploids. Neither did we see any definitive evidence of similar inversions in ABR113; higher quality reference genomes for both ABR113 and ABR114, which are in progress, will confirm or refute this finding. It is possible that the true progenitors of Bhyb26 each happened to harbor large inversions relative to all well-characterized modern *B. distachyon* and *B. stacei* lines. However, given the lack of structural variation among diverse diploids, we think a more likely explanation is that the relaxed selection accompanying WGD allowed inversions to persist in the polyploid. Whether these inversions harbor adaptive alleles, as is sometimes the case (Huang and Rieseberg 2020), will be an interesting area for future study.

Some gene loss or gain between lineages, even within the same species, is expected in the normal course of evolution (Gordon *et al.* 2017). Indeed, we observed that all our *Brachypodium* reference genomes lack at least several dozen genes that are otherwise widely conserved within and beyond the genus. However, such conspicuously absent genes were more common in the polyploids than in the diploid *Brachypodium* genomes, and they were more common in the older polyploid than the younger one. In Bhyb26, the remnants of these genes were shorter, less-expressed, and contained more premature stop codons and nonsynonymous substitutions than would be expected by random chance, suggesting that these were not real genes that were missed due to annotation error. Given that gene loss in ABR113 was greater than the sum of its progenitor species, and gene loss in Bhyb26 was greater than in ABR113, gene loss appears to be progressing gradually with time. One caveat to this analysis is that the greater gene loss in Bhyb26 could be due to demographic factors other than polyploidy. It is worth noting that our current study uncovered more potential pseudogenes in ABR113 than our previous study, likely due to our more sophisticated methods of calling synteny (Gordon *et al.* 2020).

Bhyb26 shows some evidence of post-WGD TE activity: it is slightly depleted for subgenome-specific TEs, it has more TIPs than its closest relative, and it is slightly more TE-rich than the other *Brachypodium* lineages studied here. These data are reminiscent of the *Capsella bursa-pastoris* case, in which relaxed selection permitted gradual TE proliferation following WGD (Ågren *et al.* 2016). However, we cannot exclude the possibility that these genome features were already present in the true progenitors of Bhyb26; for instance, the progenitors may have shared many TEs already at the time of WGD. Furthermore, it is possible that the inversions, gene losses, and slight TE activation are not really due to buffering by duplicate genes, but due to some demographic factor, such as a smaller population and greater genetic drift in Bhyb26 than in ABR113 for reasons other than polyploidy. Broader sampling of the D-plastotype lineage would allow for greater insight into those polyploids’ demographic histories.

It is not unusual for allopolyploids to preferentially retain genes from 1 dominant subgenome (Garsmeur *et al.* 2014; Woodhouse *et al.* 2014; Alger and Edger 2020), and it has been proposed that dominance is established immediately following WGD and increases over time (Edger *et al.* 2017). *B. hybridum* supports this model in the sense that the biased gene loss does appear to be stronger in the older lineage. However, given that the RNA-seq data do not reveal any genome dominance in either lineage, which is crucial to the proposed mechanism of genome dominance (Freeling *et al.* 2012), we cannot conclude that *B. hybridum* shows subgenome dominance in the classic sense. *B. hybridum* seems to resemble the paleoallopolyploid *Miscanthus sinensis* (Mitros *et al.* 2020) and *Cucurbita maxima* and *Cucurbita moschata* (Sun *et al.* 2017) genomes, as it is an allopolyploid that shows little to no genome dominance. Similar to cotton, our expression data are equivocal, with neither subgenome emerging as dominant across all tissues (Fang *et al.* 2017).

McClintock’s genome shock question remains a matter of much debate today: is the response to WGD predictable? Today it seems that the answer is yes and no, but our predictions are constantly becoming more sophisticated. For instance, it has been predicted that allopolyploids should show subgenome dominance over the long term (Garsmeur *et al.* 2014), but Alger and Edger (2020) and Wendel *et al.* (2018) emphasize that the key predictor of genome dominance is not necessarily progenitor divergence per se, but progenitor divergence in terms of TE load. *B. hybridum* is in line with this refined prediction, not unlike the cases of *Ephedra* (Wu *et al.* 2021) and teff (VanBuren *et al.* 2020). Genome evolution in *B. hybridum* is largely subtle and unbiased, even though it formed from a remarkably wide cross (Catalán *et al.* 2012; Dinh Thi *et al.* 2016), perhaps because its progenitors bore a similar TE load. Many genetic, epigenetic, and environmental factors contribute to a polyploid’s fate, and there is still much work to be done to determine how these factors work together. *B. hybridum* has shed some light on this complex process by providing a rare glimpse of diploidization “caught in the act.”

## Supplementary Material

iyac146_Supplementary_Data

## Data Availability

The Bhyb26 v.2.0 genome and standard annotation files are available on Phytozome. All raw sequence data used in this study, including the Bhyb26 DNA and RNA reads used for genome assembly and annotation, are available on the Joint Genome Institute Genome Portal and The National Center for Biotechnology Information Short Read Archive. Code used in this study is available at https://github.com/vtartaglio/Scarlett_et_al_2022; last accessed 6-20-2022. For accession numbers, download links, and more details on all genomes and sequencing libraries mentioned in this report, see Supplementary File 1. DOIs for the sequences produced by the JGI are: 10.46936/10.25585/60007218; 10.46936/10.25585/60001041; 10.46936/10.25585/60001092; and 10.46936/10.25585/60001143. Supplemental material is available at GENETICS online.
