# Supplementary material for: Multiple origins, one evolutionary trajectory: gradual evolution characterizes distinct lineages of allotetraploid Brachypodium
Source: Genetics. 2022 Oct 11;223(2):iyac146. doi: 10.1093/genetics/iyac146 (PMC9910409; doi:10.1093/genetics/iyac146)
Supplement: iyac146_Supplementary_Data [file iyac146_supplementary_data.zip › iyac146_Supplemental_Figure_S1.pdf]

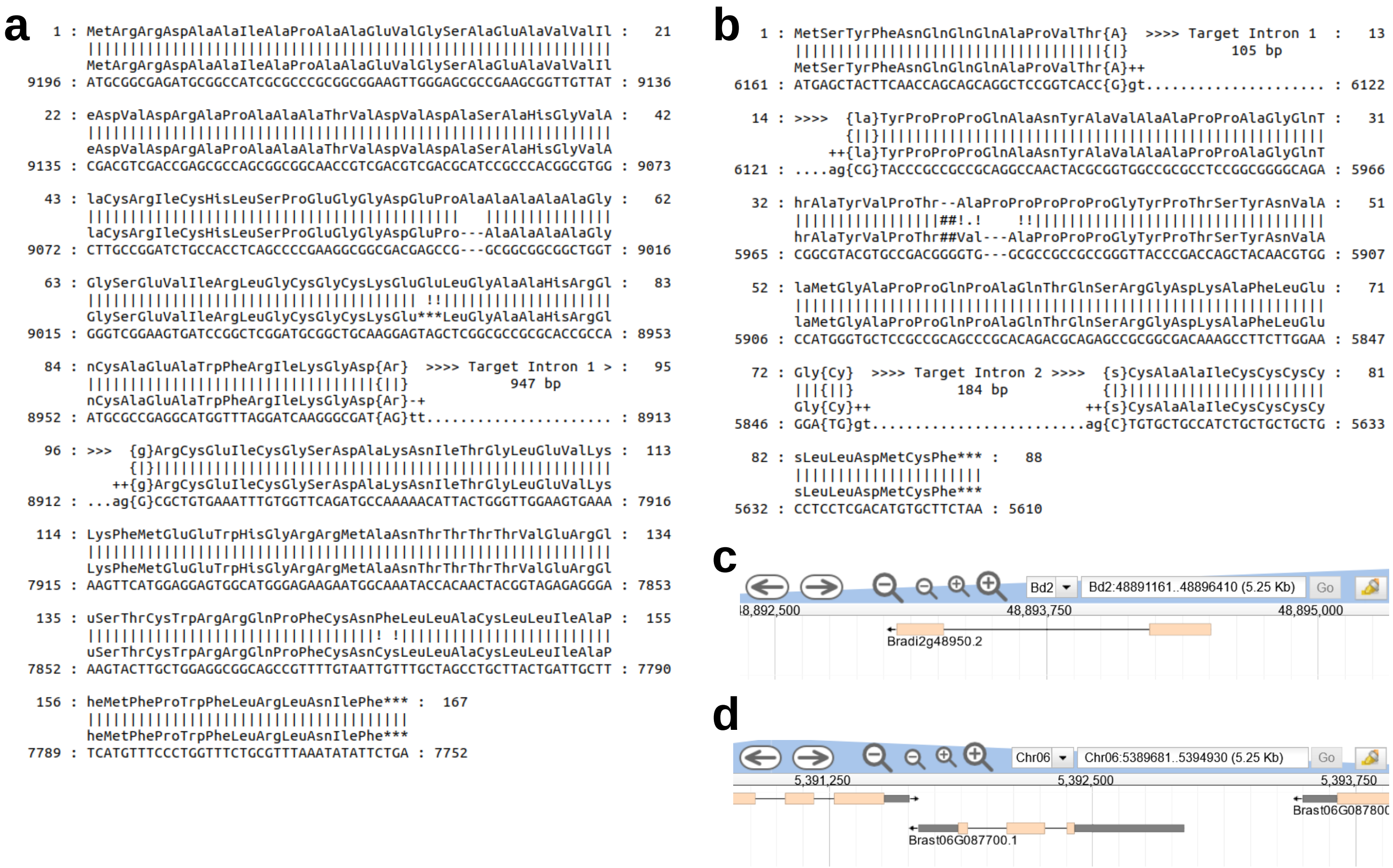

Figure S1. Example alignments of diploid peptide to polyploid genome. (a) EXONERATE alignment of the *B. distachyon* peptide for Bradi2g48950 (top) against the syntenic region in Bhyb26 (bottom), which contains a small deletion, a premature stop codon, and a substitution (F146->C). (b) EXONERATE alignment of the *B. stacei* peptide for Brast06G087700 against the syntenic region in Bhyb26, showing a more complex indel spanning about 8bp at A38. Coordinates shown correspond to site in diploid peptide or candidate Bhyb26 region, not to location in genome. (c) Gene model of Bradi2g48950 in *B. distachyon* genome. (d) Gene model of Brast06G087700 in *B. stacei* genome.
