# Supplementary material for: Multiple origins, one evolutionary trajectory: gradual evolution characterizes distinct lineages of allotetraploid Brachypodium
Source: Genetics. 2022 Oct 11;223(2):iyac146. doi: 10.1093/genetics/iyac146 (PMC9910409; doi:10.1093/genetics/iyac146)
Supplement: iyac146_Supplementary_Data [file iyac146_supplementary_data.zip › iyac146_Supplemental_Figure_S2.pdf]

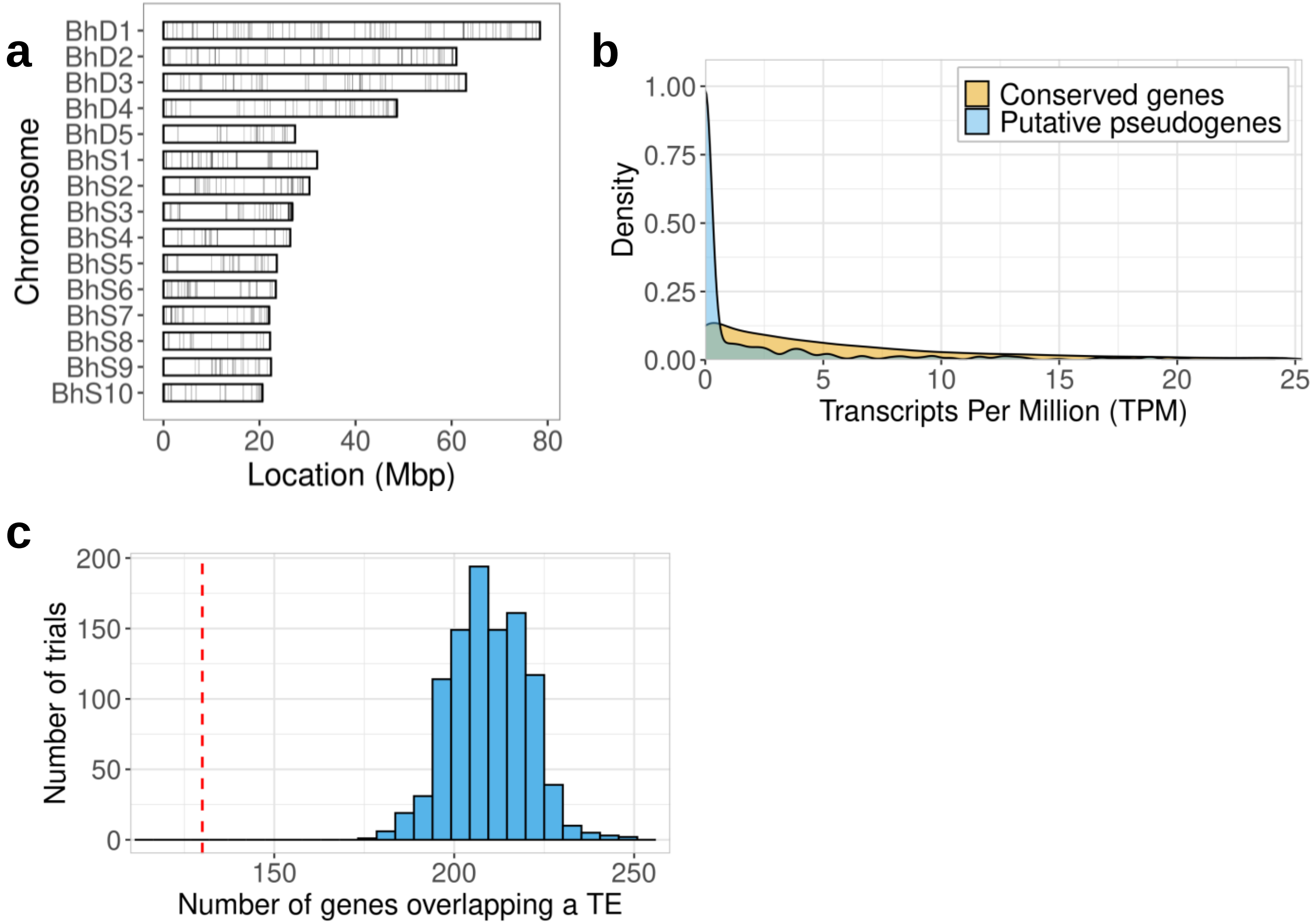

Figure S2. Characteristics of Bhyb26 candidate pseudogenes. (a) Distribution of putative pseudogenes across the Bhyb26 chromosomes. (b) The pool of Bhyb26 candidate pseudogenes is enriched for genes with low expression. Conserved gene data reflect all widely-conserved Bhyb26 genes. (c) Random samples of 464 widely-conserved genes were drawn 1,000 times, and for each trial, the number of genes overlapping a TE was recorded. 130 of the 464 putative pseudogenes overlapped a TE, indicated by the dashed red line. This was a lower rate of TE insertions than observed in any trial.
