# Supplementary material for: Multiple origins, one evolutionary trajectory: gradual evolution characterizes distinct lineages of allotetraploid Brachypodium
Source: Genetics. 2022 Oct 11;223(2):iyac146. doi: 10.1093/genetics/iyac146 (PMC9910409; doi:10.1093/genetics/iyac146)
Supplement: iyac146_Supplementary_Data [file iyac146_supplementary_data.zip › iyac146_Supplemental_Figure_S3.pdf]

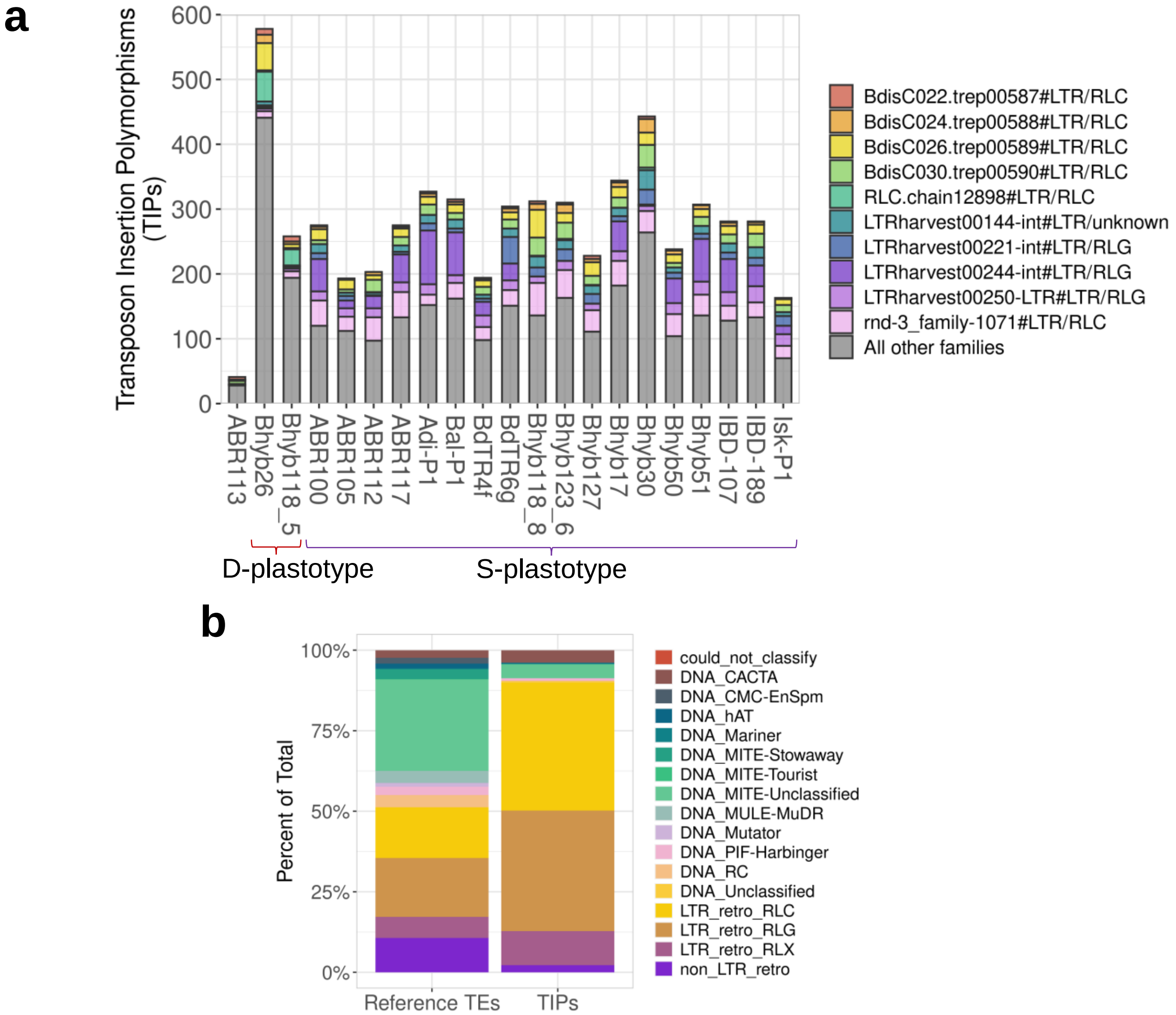

Figure S3. TE diversity *B. hybridum*. (a) Transposon Insertion Polymorphisms (TIPs) in 21 *B. hybridum* lines relative to the ABR113 genome. Colored segments show abundance of top 10 families, by median TIP contribution across lines. Gray segment shows TIPs from all other TE families. Left, ABR113 control. (b) *B. hybridum* TIP contribution disproportionately comes from *Copia* (RLC) and *Gypsy* (RLG) elements. Left, TE composition of ABR113 genome. Right, TIPs from 21 *B. hybridum* lines using ABR113 as reference.
