# Supplementary material for: Multiple origins, one evolutionary trajectory: gradual evolution characterizes distinct lineages of allotetraploid Brachypodium
Source: Genetics. 2022 Oct 11;223(2):iyac146. doi: 10.1093/genetics/iyac146 (PMC9910409; doi:10.1093/genetics/iyac146)
Supplement: iyac146_Supplementary_Data [file iyac146_supplementary_data.zip › iyac146_Supplemental_Table_S1.pdf]

Table S1. Survey of TEs in the *B. hybridum* complex.

Genotype/ subgenome	Sub/genome size (Mb)	TE space (% of genome)	Genome space occupied by DNA transposons (% of genome)	Genome space occupied by LTR retrotransposons (% of genome)	Total number of solo LTRs	Total number of full-length LTR retrotransposons	Average length of full-length LTR retrotransposons (bp)	Percent of genes that overlap a TE (%)	Percent of exons that overlap a TE (%)	Average distance from gene to nearest TE (bp) (genes that overlap a TE excluded)
Bd21	275	29.0	6.5	22.5	2,244	185	4,706	41.4	2.2%	1462
ABR114	234	20.4	5.5	14.9	1,259	64	4,663	42.0	3.3%	1276
ABR113-D	269	27.8	6.0	20.3	3,087	125	4,864	40.6	5.8%	1334
ABR113-S	240	22.2	5.2	15.6	1,973	108	5,636	43.0	4.4%	1235
Bhyb26-D	278	30.6	6.5	22.1	3,625	137	4,888	41.7	2.2%	1304
Bhyb26-S	249	23.9	5.4	16.8	3,250	122	5,116	43.7	2.1%	1237
